# Genome Sequence of *Deinococcus marmoris* PAMC 26562 Isolated from Antarctic Lichen

**DOI:** 10.1128/genomeA.00013-17

**Published:** 2017-03-23

**Authors:** Junghee Kim, Kae Kyoung Kwon, Byung Kwon Kim, Soon Gyu Hong, Hyun-Myung Oh

**Affiliations:** a Department of Marine-Bio Convergence Science, Specialized Graduate School Science & Technology Convergence, Pukyong National University, Nam-gu, Busan, Republic of Korea; b Marine Biotechnology Research Division, Korea Institute of Ocean Science & Technology, Ansan, Republic of Korea; c OmicsPia Co., Ltd., Daejeon, Republic of Korea; d Division of Life Sciences, Korea Polar Research Institute, Incheon, Republic of Korea

## Abstract

*Deinococcus marmoris* strain PAMC 26562 was isolated from *Usnea* sp., a lichen collected from King George Island, Antarctica. We report here the draft genome sequence of strain PAMC 26562, which has xanthorhodopsin and carbon monoxide dehydrogenase genes in addition to major metabolic pathways presented in deinococcal genomes.

## GENOME ANNOUNCEMENT

The phylum *Deinococcus-Thermus* constitutes one of the major bacterial evolutionary lineages (1). Species of the genus *Deinococcus* are strictly aerobic, have optimum growth temperatures in the range from 25 to 35°C, produce reddish colonies, generally stain Gram positive, have ornithine in the peptidoglycan, lack teichoic acids, possess menaquinone 8 as the major respiratory quinone, and are phylogenetically related to the thermophilic species of the genera *Thermus* and *Meiothermus* (2). Most members of the genus *Deinococcus* are able to grow in the presence of high levels of chronic radiation toxicity and desiccation, because they can protect enzymes from reactive oxygen species generated during ionizing radiation (3). In a previous study, a *Deinococcus* strain had been found to be associated with an *Usnea* species (4). The strain PAMC 26562 was deposited under Polar and Alpine Microbial Collection (PAMC) project carried out by the Korean Polar Research Institute (KOPRI) (5). The closest known species to strain PAMC 26562 was *Deinococcus marmoris*, based on the 16S rRNA sequence analysis (accession number AJ585986) (4).

Here, we report the draft genome sequence of the strain PAMC 26562. Strain PAMC 26562 was isolated from a lichen collected from King George Island, Antarctica (4). The draft genome sequence was determined using the Illumina sequencing, and assembly program used was Ray (version 2.3.1) (6). Assembly of genome analysis was carried out with increasing *k*-mer values from 5 to 65. The longest *N*_50_ and contigs were reached when the *k*-mer was 27. Bowtie-2 (version 2.2.6) was used to generated a *.sam file (7), and SAMtools (version 0.1.19) was used to generated a *.bam file (8). BEDTools (bedtools genomecov; aka “genomeCoverageBed” version 2.25.0) was used to calculate genome coverage (9); Illumina reads aligned in a *.bam file corresponded to 121× coverage depth. Finally, 187 contigs were submitted to GenBank. Three contigs with no coding sequence (CDS) features and 30 contigs less than 500 bp in length were excluded. The draft genome of PAMC 26562 comprised 5,286,835 bp, with a G+C content of 64.1%.

There were 5,027 protein-coding sequences in total, and they were annotated by the RAST server (10), according to a previously described method (11). Some genes of interest were confirmed using blast using National Center for Biotechnology Information (NCBI) database. Noncoding genes included six 16S rRNA genes and 49 tRNA-coding genes. By using the CRISPRFinder program analysis (12), six contigs from the strain PAMC 26562 had hits to a clustered regularly interspaced short palindromic repeat (CRISPR) sequence. In addition to common metabolism and stress responses, like radiation and desiccation, xanthorhodopsin/retinoid synthesis and carbon monoxide dehydrogenase gene clusters could be found, as in the case with some other genomes in the genus *Deinococcus*.

### Accession number(s).

This whole-genome shotgun project for *Deinococcus marmoris* strain PAMC 26562 has been deposited at DDBJ/ENA/GenBank under the accession no. MSTI00000000. The version described in this paper is version MSTI01000000.
